# Ultrasonic and Resistivity Sensor Fusion for Discriminating Gas Injection Rates

**DOI:** 10.3390/s26185749

**Published:** 2026-09-10

**Authors:** Juehui Wang, Xiuquan Li, Gui Tang, Yilin Yao, Qiuyue Wang, Weijie Li, Mingzhang Luo

**Affiliations:** 1 School of Electronic Information and Electrical Engineering, Yangtze University, Jingzhou 434023, China; wangjh.st@yangtzeu.edu.cn (J.W.); qiuyue.st@yangtzeu.edu.cn (Q.W.); 2 Drilling and Production Engineering Technology Research Institute, CNPC Chuanqing Drilling Engineering Co., Ltd., Guanghan 618300, China; tangg_ccde@cnpc.com.cn (G.T.); yaoyili_ccde@cnpc.com.cn (Y.Y.); 3 School of Civil Engineering, Dalian University of Technology, Dalian 116024, China; liweijie@dlut.edu.cn

**Keywords:** two-phase flow, ultrasonic transmission, resistivity sensing, feature extraction, sensor fusion, sliding window

## Abstract

Discriminating gas injection rates in drilling fluids is essential for monitoring downhole gas influx. However, each gas bubble traversing the sensing zone causes ultrasonic and resistivity readings to depart from the baselines recorded without gas injection, while a single acquisition cycle captures only a random snapshot of the bubble distribution. In this work, a sliding window framework is developed that combines ultrasonic and resistivity measurements to distinguish gas injection rates in a vertical static liquid column. Ultrasonic and resistivity indices are extracted from individual acquisition cycles, aggregated within a 2.5 s sliding window containing five consecutive acquisition cycles, and fused by normalizing classwise products without introducing trainable parameters. Experiments were conducted in freshwater and $2.0\;g\;{\mathrm{cm}}^{-3}$ water-based mud at four discrete injection rates from 0 to $0.3\;L\;\min^{-1}$. Evaluation used cross-validation with data split by experimental repetition. The fusion framework achieved a macro F1 of 84.7% ± 4.3% in water-based mud, a gain of 6.3 percentage points over the best result from either channel alone. The classwise product also gave the highest mean macro F1 among three evaluated nontrainable fusion rules in both media. The resulting fusion framework supports gas injection rate discrimination under static column conditions.

## 1. Introduction

In drilling operations, formation gas influx into the wellbore can lead to a blowout, making early gas kick detection essential for well control safety [1,2,3]. Direct downhole measurement of physical contrast changes in the gas–liquid medium bypasses the transport delay inherent in surface detection [4,5]. Physics informed approaches have been applied to quantitative interpretation of gas kicks [6]. However, the downhole environment poses inherent challenges. Opaque drilling fluids impede optical instrumentation, while suspended solid particles in mud scatter ultrasonic waves, reducing penetration depth. Electrical measurements can also be affected by contact impedance and electrode polarization [7,8]. In bubbly liquids, surface active additives can further alter the bubble size distribution that any single sensor must resolve [9]. Single sensors cannot maintain stable discrimination under randomly fluctuating bubble distributions. Multisensor fusion is therefore a well-motivated research direction.

Multisensor combinations expand information coverage but do not eliminate single cycle fluctuations caused by intermittent bubble passage. In gas–liquid two-phase flow, instantaneous sensor readings are primarily determined by the local bubble distribution. Variations in bubble size further increase uncertainty in ultrasonic measurements [10]. At low injection rates, the sensing zone experiences extended periods without bubbles, whereas at high rates, bubbles pass almost continuously. This instability in the relationship between instantaneous local readings and the macroscopic injection rate represents a central difficulty in two-phase flow measurement. Most established approaches have focused on sensor selection or single feature design, with limited attention to bubble intermittency as a primary factor in measurement strategy design.

On the ultrasonic side, transmission and reflection methods have been widely applied to two-phase flow measurement [11,12]. Multisensor configurations, including acoustic optical Venturi combinations [13] and ultrasonic tomography [14], have been used for void fraction prediction and bubble imaging. Ultrasonic phased arrays [15] provide additional spatial resolution. Quantitative acoustic methods [16,17] have also been applied to gas kick identification. In mud, solid phase particles further scatter and attenuate ultrasonic signals, increasingly complicating their interpretation. On the electrical side, conductance and impedance sensors have been employed for phase fraction measurement and flow pattern identification [18,19]. Combinations of electrical resistance tomography, electrical capacitance tomography, and a Venturi tube [20], as well as capacitively coupled electrical impedance imaging [21], have increased the dimensionality of the measurements.

On the fusion side, multisensor statistical approaches include a dual sensor method combining a Venturi tube and a microwave resonator [22], and spatiotemporal feature fusion for flow pattern recognition [23]. Fusion of multiple near-infrared channels [24] and fusion based on machine learning [25] have also been applied to two-phase flow measurement. Multisensor fusion approaches have been further surveyed in the context of machine learning integration with multiphase flow sensors [26], and soft computing models for mass flow rate measurement [27]. These works combine signals from multiple sources. When applied to intermittent bubble passage, however, existing fusion methods either rely on time-averaged quantities that sacrifice temporal resolution [22], or require substantial training data for feature extraction [23].

Combinations of electrical and ultrasonic sensing have been explored for two-phase flow imaging [28,29] and for inline monitoring of drilling returns [30], but their application to gas influx discrimination through interpretable fusion has not been reported. Using controlled gas injection to emulate the gas–liquid state associated with an incipient gas influx, this work proposes a framework that combines ultrasonic and resistivity measurements with temporal aggregation in a sliding window to discriminate the imposed gas injection rates in a vertical static liquid column. The Ultrasonic Propagation Index (UPI) combines relative changes in TOF, amplitude, and waveform correlation. Relative Resistivity Change (RRC) measures the relative change in the digitized electrical response. The ultrasonic response is averaged along the propagation path, whereas the electrical response is weighted by the current field around the probe. Their complementary spatial sensitivities provide the basis for combining the two channels. Preliminary decisions from individual acquisition cycles are aggregated within a sliding window, and the resulting class frequencies are fused by normalizing classwise products. The framework is evaluated at gas injection rates of 0, 0.1, 0.2, and $0.3\;L\;\min^{-1}$.

## 2. Materials and Methods

### 2.1. Sensing Principles

**Figure 1:**
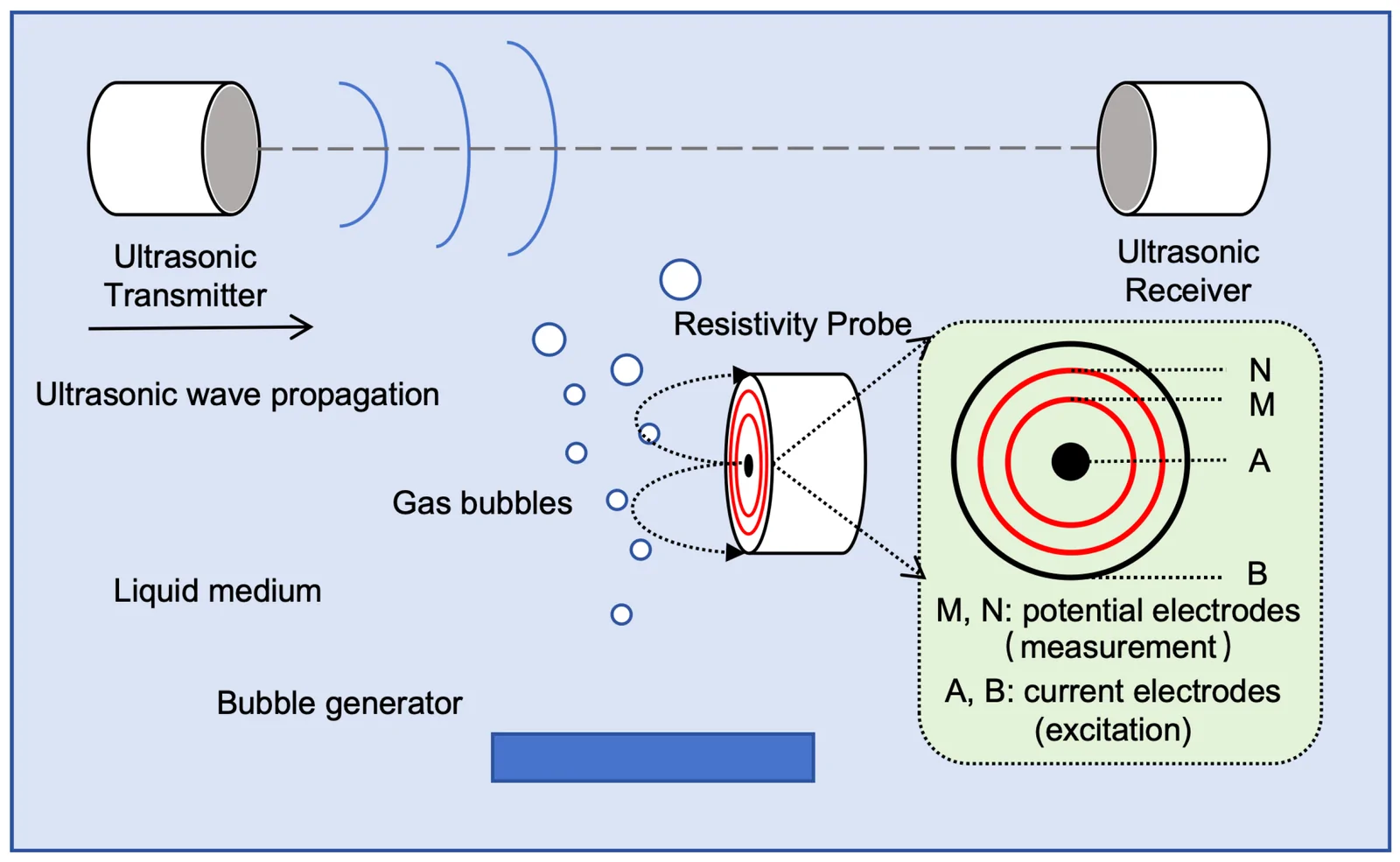
Sensing principles of the ultrasonic transmission channel and the resistivity probe. Both sensors monitor the same vertical section of the static column but respond over different spatial regions.

The sensing framework combines ultrasonic transmission with a resistivity probe to characterize bubble passage through the same vertical section of the static column. The ultrasonic channel responds to changes along the propagation path, whereas the resistivity probe is primarily sensitive to the current field near the electrodes. Figure 1 illustrates these complementary sensing regions.

Gas bubbles alter the effective density and compressibility of the liquid medium. For an idealized bubbly mixture, the Wood relation gives the effective density $\rho_{\mathrm{eff}}$ and sound speed $c_{\mathrm{eff}}$ in terms of the local gas volume fraction α [31,32].

(1) $$\frac{1}{\rho_{\mathrm{eff}}c_{\mathrm{eff}}^{2}}=\frac{\alpha }{\rho_{g}c_{g}^{2}}+\frac{1-\alpha }{\rho_{l}c_{l}^{2}}$$

The subscripts *g* and *l* denote the gas and liquid phases. Increasing α reduces the effective sound speed and changes the time of flight (TOF) over a fixed propagation path. Bubble scattering and attenuation can also alter the received amplitude and waveform shape [10]. These responses motivate the use of TOF, amplitude, and waveform correlation in the ultrasonic feature.

Bubbles in the conductive liquid act as a nonconducting phase. For a dilute dispersion, the Maxwell relation gives the effective resistivity $\varrho_{\mathrm{eff}}$ relative to the resistivity $\varrho_{l}$ of the liquid phase [33].

(2) $$\varrho_{\mathrm{eff}}=\varrho_{l}\frac{2+\alpha }{2(1-\alpha )}$$

Increasing the local gas fraction therefore increases the effective resistivity, providing a physical basis for detecting bubble passage with the resistivity probe. The probe senses this change with four electrodes. A voltage pulse excites the outermost ring and the central disk, which inject current into the liquid. The resulting potential is measured across the two intermediate rings. The digitized response is a relative electrical measurement rather than an absolute resistivity inversion. Relative Resistivity Change (RRC) quantifies its deviation from the reference cycle.

Because the two sensing channels respond over different spatial regions, their changes need not coincide in every acquisition cycle. Increasing the imposed gas injection rate changes the occurrence and persistence of these local responses. The features extracted from individual acquisition cycles are therefore aggregated within a sliding window before the two channels are combined.

### 2.2. Experimental Setup

**Figure 2:**
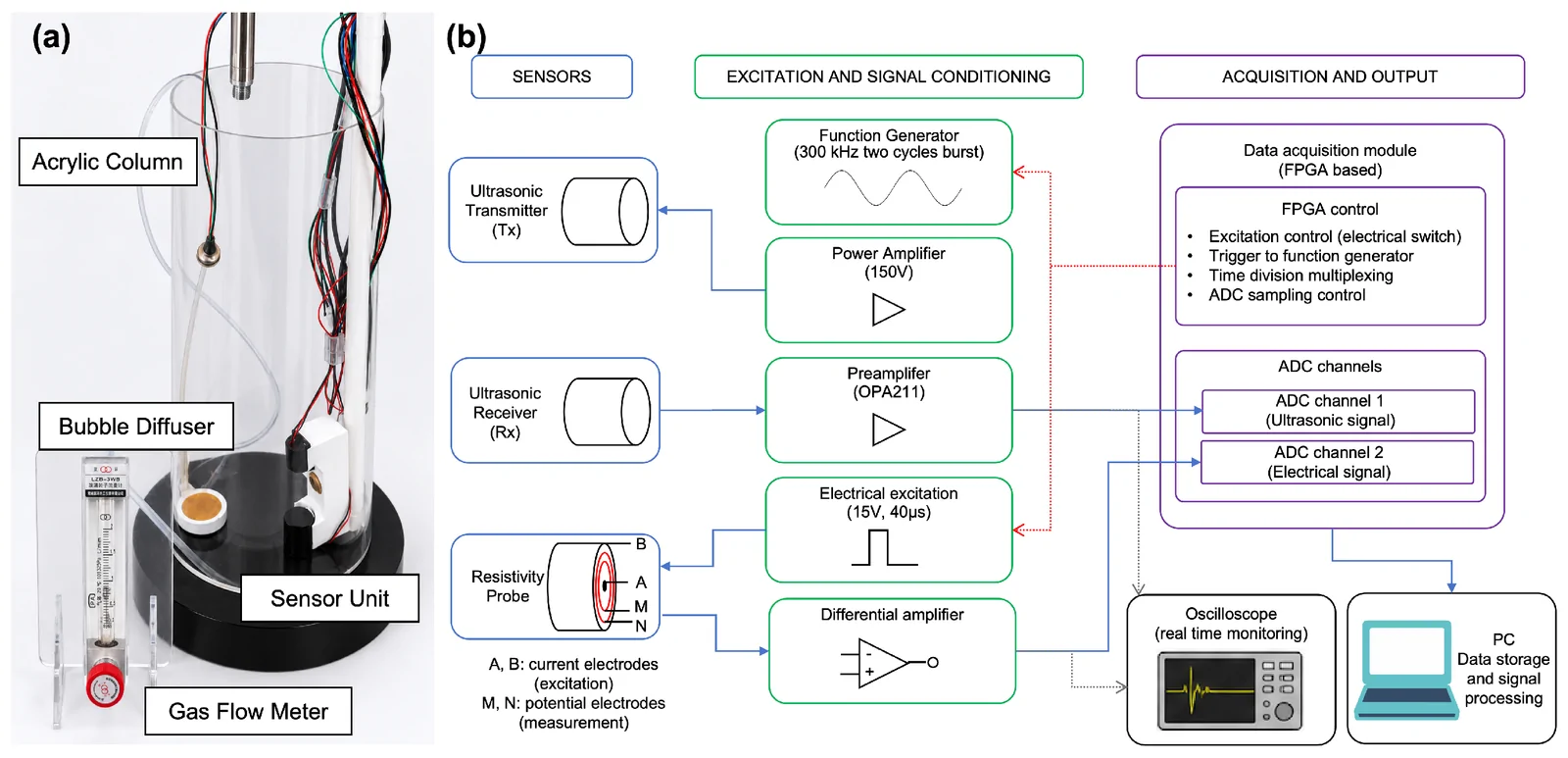
Experimental setup. (**a**) Test column with the gas flow meter and sensor mounting. (**b**) Schematic diagram of the electronic control and acquisition system.

The experimental setup is shown in Figure 2. The test column in Figure 2a is a transparent acrylic cylinder with an inner diameter of 22cm and a height of 50cm. It was filled either with freshwater or with water-based mud of density $2.0\;g\;{\mathrm{cm}}^{-3}$. A gas injection control unit consisting of a pump, a gas flow meter, and a gas generator delivered rates from 0 to $0.3\;L\;\min^{-1}$.

A pair of ultrasonic transducers with a center frequency of 300kHz was mounted on opposite sides of the column for transmission measurements. The transmitter was driven by a 150V rectangular pulse train containing two cycles at 300kHz, generated by a function generator followed by a custom-built power amplifier. The received signal was conditioned by a custom preamplifier using an OPA211 (Texas Instruments, Dallas, TX, USA) with a gain of 6dB and a bandpass filter from 200 to 350kHz. A concentric ring resistivity probe with four electrodes was mounted adjacent to the ultrasonic transducers and driven by a voltage pulse. The probe face had a diameter of 18.0mm and consisted of a central disk electrode surrounded by three concentric copper rings with gold plating. The spacing between rings was 1.0mm and each electrode was 1.2mm wide.

The electronic control and acquisition units in Figure 2b included a COMPACT-TCP-4-250M acquisition card (Wuhan Compact Technology Co., Ltd., Wuhan, China) with four channels and 12 bit resolution. The acquisition card used an FPGA to control the excitation timing and ADC sampling through time division multiplexing, with an effective sampling rate of 10MHz. The ultrasonic preamplifier output and the electrical differential amplifier output were acquired through two of these channels. The computer stored and processed the acquired data, while the oscilloscope monitored the two conditioned signals during the experiment. The 3D printed fixtures in Figure 2a secured both sensing modules above the gas injection point so that they monitored the same vertical section of the column.

### 2.3. Experimental Procedure

Experiments were conducted in the static column without circulating flow to minimize pressure fluctuations and mechanical vibration. Before gas injection, the column was filled with the working fluid and left undisturbed for 3 h to allow residual bubbles to dissipate and the fluid temperature to stabilize. A 30 min continuous recording was then obtained without gas injection. For each medium, one acquisition cycle from the final 10 min was selected as the fixed reference cycle used throughout subsequent processing.

Air was injected at four discrete rates of 0, 0.1, 0.2, and $0.3\;L\;\min^{-1}$ in ascending order. The gas flow meter was used to set each rate. The observed bubble diameters were approximately 1–5mm. A stabilization period of at least 10 min followed each adjustment, after which data were extracted from the steady-state segment. The sequence was repeated three times for each medium in separate time slots. The condition without gas injection was reestablished before every repetition. Each rate provided 204 acquisition cycles for each medium, with 68 cycles from each repetition.

The two channels monitored the same vertical section by time division multiplexing at two acquisition cycles per second. The electrical measurement was performed first in each cycle. A 15V pulse with a duration of 40μs excited the outermost ring and the central disk, which served as the current electrodes. The voltage across the two intermediate rings, which served as the potential electrodes, was digitized at 10MHz for 1024 sample points. After a hardware delay of 50μs, the ultrasonic transmitter emitted the pulse train and the received waveform was sampled at the same rate and length. Each acquisition cycle therefore contained one digitized electrical response and one received ultrasonic waveform.

### 2.4. Feature Extraction

**Figure 3:**
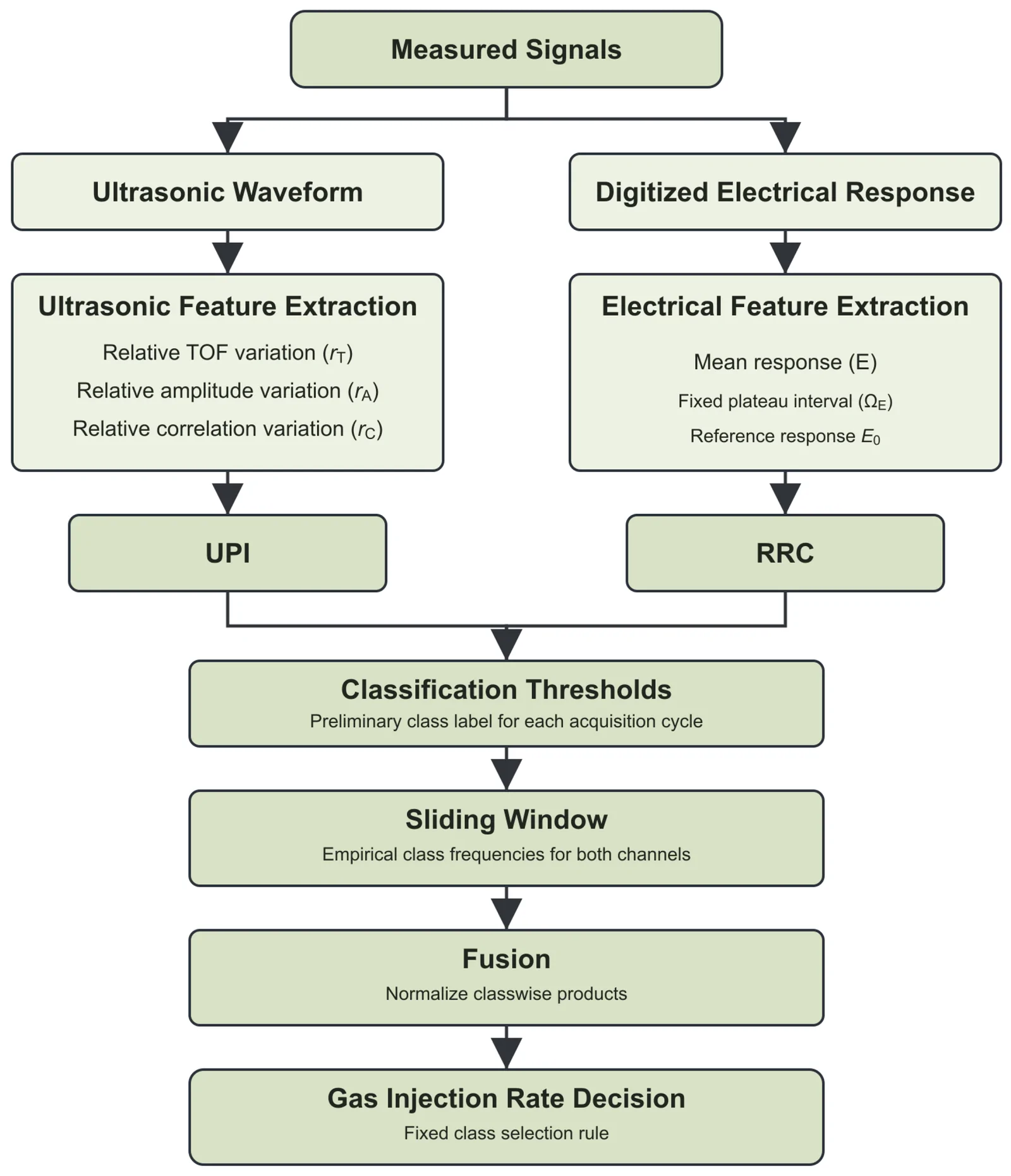
Signal processing pipeline. Relative variations in ultrasonic TOF, amplitude, and waveform correlation form the Ultrasonic Propagation Index (UPI). The mean digitized electrical response forms the Relative Resistivity Change (RRC). Classification thresholds, sliding window frequencies, and fusion by normalizing classwise products then produce the gas injection rate decision.

Figure 3 summarizes the processing sequence from the two measured signals to the final gas injection rate decision. A fourth order Butterworth bandpass filter from 50 to 600kHz was applied to the received ultrasonic waveforms in both forward and reverse directions. For each medium, the fixed reference cycle provided a received waveform $x_{0}\left[p\right]$, from which a reference segment $s_{0}\left[m\right]$ was extracted.

For each received ultrasonic waveform $x_{n}\left[p\right]$ from acquisition cycle *n*, the reference segment was compared with candidate segments in the prescribed search region. Both segments were standardized by subtracting their means and dividing by their standard deviations before the discrete cross-correlation was calculated as

(3) $$R_{n}\left(k\right)=\sum_{m=0}^{M-1}\frac{s_{0}\left[m\right]-{\bar{s}}_{0}}{\sigma_{s_{0}}}\frac{x_{n}\left[k+m\right]-{\bar{x}}_{n,k}}{\sigma_{x,n,k}},$$

where *M* is the length of the reference segment and *k* is the starting sample of the candidate segment. The position of the maximum cross-correlation was used as the estimated TOF $T_{n}$, and the maximum value was retained as $C_{n}$ [34]. The search for each received waveform was constrained to within 10% of the reference TOF position. The reference values $T_{0}$ and $C_{0}$ were calculated from the fixed reference cycle.

The received amplitude was extracted from a fixed interval selected for each medium. Among the first six detected positive peaks, the two largest were identified and the earlier one was used as the amplitude $A_{n}$. The same rule was applied to the reference waveform to obtain $A_{0}$. The relative variations in amplitude, TOF, and waveform correlation were calculated and combined to form the UPI as

(4) $$\begin{array}{cc} r_{A,n} & =\left|\frac{A_{n}-A_{0}}{A_{0}}\right|\times 100,\\ r_{T,n} & =\left|\frac{T_{n}-T_{0}}{T_{0}}\right|\times 100,\\ r_{C,n} & =\left|\frac{C_{n}-C_{0}}{C_{0}}\right|\times 100,\\ {\mathrm{UPI}}_{n} & =\frac{r_{A,n}}{\left(1-r_{T,n}/100\right)\left(1-r_{C,n}/100\right)}. \end{array}$$

The UPI is an empirical feature in which the amplitude variation forms the primary term, while the normalized changes in TOF and waveform correlation provide monotonic modulation within the observed feature ranges. This fixed form retains an explicit relationship among the three waveform changes without introducing fitted feature weights.

The resistivity channel was processed independently. For acquisition cycle *n*, $v_{n}\left[p\right]$ denotes the digitized voltage at sample *p*, and the mean response $E_{n}$ was calculated over a fixed interval $\Omega_{E}$ on the response plateau.

(5) $$E_{n}=\frac{1}{|\Omega_{E}|}\sum_{p\in \Omega_{E}}v_{n}\left[p\right],$$

Applying the same calculation to the fixed reference cycle produced the reference electrical response $E_{0}$. Relative Resistivity Change (RRC) was calculated from the digitized response of the resistivity probe relative to this reference.

(6) $${\mathrm{RRC}}_{n}=\left|\frac{E_{n}-E_{0}}{E_{0}}\right|\times 100.$$

### 2.5. Window Fusion

Classification thresholds mapped each UPI and RRC value from an individual acquisition cycle to one of four preliminary classes. The class index set I={1,2,3,4} corresponds in order to gas injection rates of 0, 0.1, 0.2, and $0.3\;L\;\min^{-1}$. For a window of length *W* ending at cycle *t*, the empirical class frequencies for the ultrasonic and resistivity channels were

(7) $$P_{U}^{\left(i\right)}\left(t\right)=\frac{n_{U}^{\left(i\right)}\left(t\right)}{W},\;P_{R}^{\left(i\right)}\left(t\right)=\frac{n_{R}^{\left(i\right)}\left(t\right)}{W},\;i\in I,$$

where $n_{U}^{\left(i\right)}$ and $n_{R}^{\left(i\right)}$ are the numbers of cycles assigned to class *i* within the window. These quantities are empirical frequencies. The two frequency vectors were fused by normalizing their classwise products.

(8) $$\begin{array}{cc} q_{i}\left(t\right) & =P_{U}^{\left(i\right)}\left(t\right)P_{R}^{\left(i\right)}\left(t\right),\\ S_{i}\left(t\right) & =\frac{q_{i}\left(t\right)}{\sum_{j\in I}q_{j}\left(t\right)}. \end{array}$$

If all classwise products were zero, unweighted averaging was used instead, with $S_{i}\left(t\right)=\left[P_{U}^{\left(i\right)}\left(t\right)+P_{R}^{\left(i\right)}\left(t\right)\right]/2$. A class selection threshold of 0.4 was used for final class selection. Let $V\left(t\right)=\{i\in I:S_{i}\left(t\right)\geq 0.4\}$. The predicted class was

(9) $$\hat{y}\left(t\right)=\left\{\begin{array}{cc} \max V(t), & V(t)\neq ⌀,\\ \arg\max_{i\in I}S_{i}\left(t\right), & V(t)=⌀. \end{array}\right.$$

**Figure 4:**
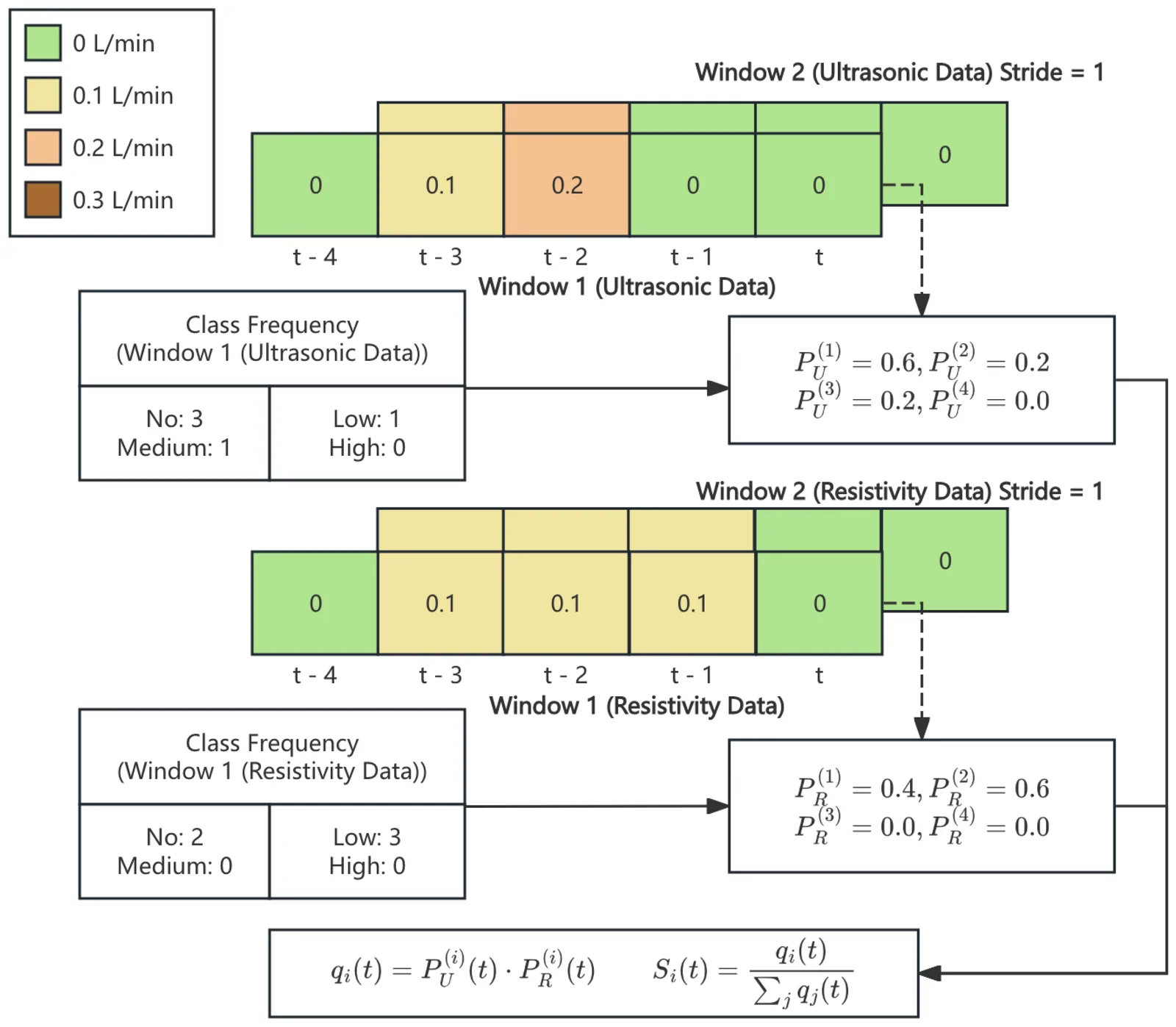
Fusion using a sliding window. Empirical class frequencies from both sensing channels are combined by normalizing classwise products.

The same selection rule was used when evaluating either sensing channel alone. The sliding window was set to 2.5 s, corresponding to five consecutive acquisition cycles, and was advanced by one cycle for each decision. Successive outputs were therefore separated by 0.5 s. Windows were confined to individual experimental repetitions. Figure 4 illustrates one update of the class frequencies and fusion scores.

### 2.6. Evaluation Procedure

Evaluation used cross-validation with data split by experimental repetition [35]. Across the three folds, each complete repetition containing all four injection rates was reserved once for evaluation, while the other two repetitions were used to select the UPI and RRC thresholds. Thresholds were selected independently for the two channels from predefined candidate grids by maximizing the macro F1 score over those two repetitions. The selected thresholds were fixed before they were applied to the reserved repetition. The reference cycle and feature extraction settings were also fixed for each medium before evaluation. The predefined candidate grids covered the expected boundary intervals between adjacent gas injection rate classes, and in each fold threshold selection used only the two repetitions assigned to threshold selection. Freshwater and mud were evaluated separately.

Each repetition contained 68 acquisition cycles and yielded 64 overlapping windows, giving 192 windows per rate. Table 1 reports the ranges of thresholds selected across the three folds.

Performance was evaluated for binary detection of gas injection and for discrimination among the four injection rates. For binary detection, No Injection was treated as the negative class and the three nonzero rates as the positive class. The false alarm and miss rates were obtained from the confusion matrices pooled across the three reserved repetitions, while the binary F1 score was calculated per reserved repetition. For discrimination among the four injection rates, precision, recall, and F1 score were calculated for each class, and the macro F1 score was taken as their unweighted mean. The two F1 scores were reported as the mean ± standard deviation across the three folds.

The primary evaluation used W=5. Window length sensitivity was assessed at W=1,3,5,7,9, and 11. An ablation analysis compared configurations that isolated the contributions of window aggregation and fusion of the two channels. For configurations based on individual acquisition cycles, decisions were evaluated at the same 64 positions per repetition used by the W=5 configurations. This protocol for matching decision positions differs from the W=1 sensitivity evaluation, which used all 68 acquisition cycles in each repetition.

Sensitivity to the fusion rule was evaluated under the same cross-validation folds, channel thresholds, and W=5 windows. The classwise product was compared with unweighted averaging, $S_{i}^{\mathrm{avg}}=\left[P_{U}^{\left(i\right)}+P_{R}^{\left(i\right)}\right]/2$, and an adapted Noisy-OR rule, ${\tilde{S}}_{i}^{\mathrm{NOR}}=1-\left[1-P_{U}^{\left(i\right)}\right]\left[1-P_{R}^{\left(i\right)}\right]$ [36]. The Noisy-OR scores were normalized across the four classes before the final selection rule in Equation (9) was applied.

## 3. Results and Discussion

### 3.1. Sensor Response Characteristics

**Figure 5:**
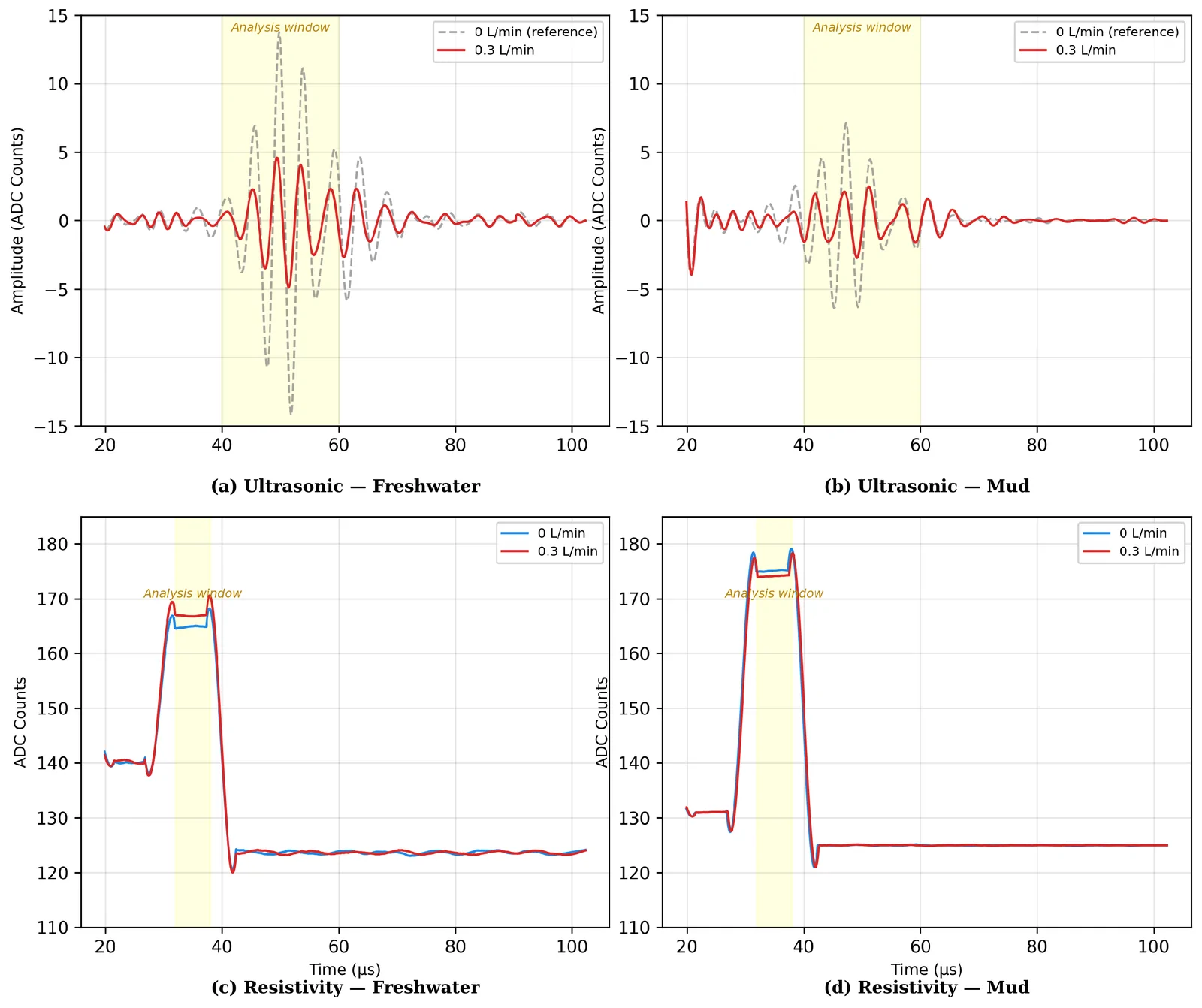
Example received ultrasonic waveforms and digitized electrical responses from individual acquisition cycles at 0 and $0.3\;L\;\min^{-1}$ in freshwater and $2.0\;g\;{\mathrm{cm}}^{-3}$ water-based mud.

**Figure 6:**
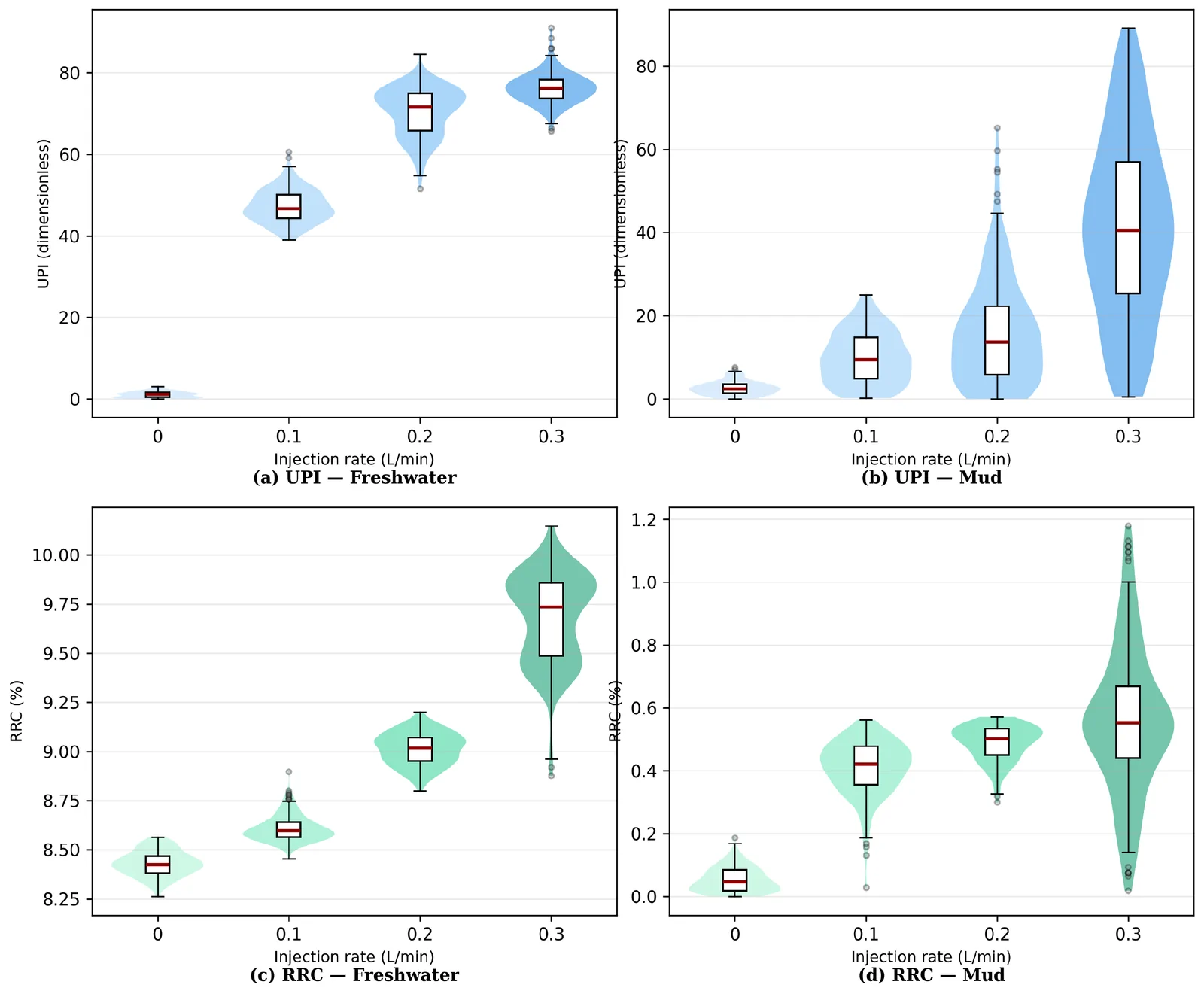
Distributions of UPI and RRC from individual acquisition cycles in freshwater and $2.0\;g\;{\mathrm{cm}}^{-3}$ water-based mud. The violin plots are overlaid with box plots showing the median, interquartile range, and 1.5 interquartile range whiskers.

Figure 5 presents example responses from individual acquisition cycles at 0 and $0.3\;L\;\min^{-1}$. The principal ultrasonic wave packets occur within similar time intervals in the displayed traces, while their peak amplitudes decrease from 14 to 5 ADC counts in freshwater and from 7 to 3 counts in mud. The digitized electrical response has a rectangular pulse shape with a nearly flat plateau, whose level rises slightly at $0.3\;L\;\min^{-1}$ in both media. These records show the signal morphology in individual acquisition cycles, while variability across all cycles is examined through the feature distributions in Figure 6.

Adjacent class separation was quantified using the Fisher discriminant ratio (Table 2), $J_{ij}={\left(\mu_{i}-\mu_{j}\right)}^{2}/\left(\sigma_{i}^{2}+\sigma_{j}^{2}\right)$, where μ and $\sigma^{2}$ are the mean and variance of the cycle level feature values. Lower values at the main difficult boundaries support the overlap visible in Figure 6, although the ratios do not correspond directly to class F1 values.

In freshwater, RRC medians rise monotonically from 8.42% at baseline to 9.75% at the highest rate (Figure 6c). The interquartile ranges are narrow and shift upward with injection rate, while the No Injection and 0.1 L min^−1^ distributions overlap slightly at their tails. The ultrasonic response also increases with injection rate (Figure 6a), but the median shift is smaller at the two upper rates. Their distributions overlap substantially, indicating reduced resolution between 0.2 and 0.3 L min^−1^.

In 2.0 g cm^−3^ water-based mud, the response pattern differs from that in freshwater. UPI medians at 0.1 and 0.2 L min^−1^ remain below 20 (Figure 6b). At 0.3 L min^−1^, the median rises to 40.57 and the distribution broadens across much of the observed range. RRC rises from a baseline median of 0.05% to 0.42% at 0.1 L min^−1^, with smaller median changes at the two upper rates (Figure 6d). The broad 0.3 L min^−1^ distribution encompasses the narrower distribution at 0.2 L min^−1^. No single channel therefore separates all four rates in both media.

The broad and overlapping distributions may reflect changes in the local bubble distribution from one acquisition cycle to another. Because each cycle samples the sensing zone at one instant, different bubble configurations can produce different feature values at the same imposed rate. Shifts in the medians with rate therefore coexist with substantial variability and overlap between individual cycles.

The contrast between the two channels may partly reflect the spatial sensitivities described in Section 2.1. The ultrasonic measurement represents propagation along the beam path, whereas the electrical response is weighted toward the region around the probe electrodes. These sensitivities provide a physical basis for complementary responses, but the present measurements do not isolate the causes of the smaller response changes at the upper rates. Independent measurements of the local bubble distribution, using a void fraction reference such as a wire mesh sensor, are needed to test this interpretation [37].

### 3.2. Binary Gas Detection

Table 3 reports performance for distinguishing any gas injection from the No Injection baseline. The binary results reflect the response patterns shown in Figure 6.

In freshwater, the ultrasonic channel detects all injection states without error, yielding a binary F1 score of 100.0%. The resistivity channel alone generates 34 false alarms across 192 baseline windows. The overlap at the tails of the No Injection and 0.1 L min^−1^ distributions in Figure 6c may contribute to this 17.7% false alarm rate. Fusion matches the ultrasonic result and produces no baseline false alarms.

In mud, the resistivity channel achieves a binary F1 score of 99.8%, while the ultrasonic channel yields 94.9%. Fusion produces no baseline false alarms but misses nine windows with gas injection, including two at 0.2 and seven at 0.3 L min^−1^. Its binary F1 score of 99.2% therefore remains slightly below the resistivity result.

The binary F1 scores range from 94.9% to 100.0% across the channel and medium combinations. Fusion matches the best individual channel in freshwater but does not improve on RRC in mud. Discrimination among the four injection rates therefore provides a more demanding evaluation of the combined measurements.

### 3.3. Injection Rate Discrimination

**Figure 7:**
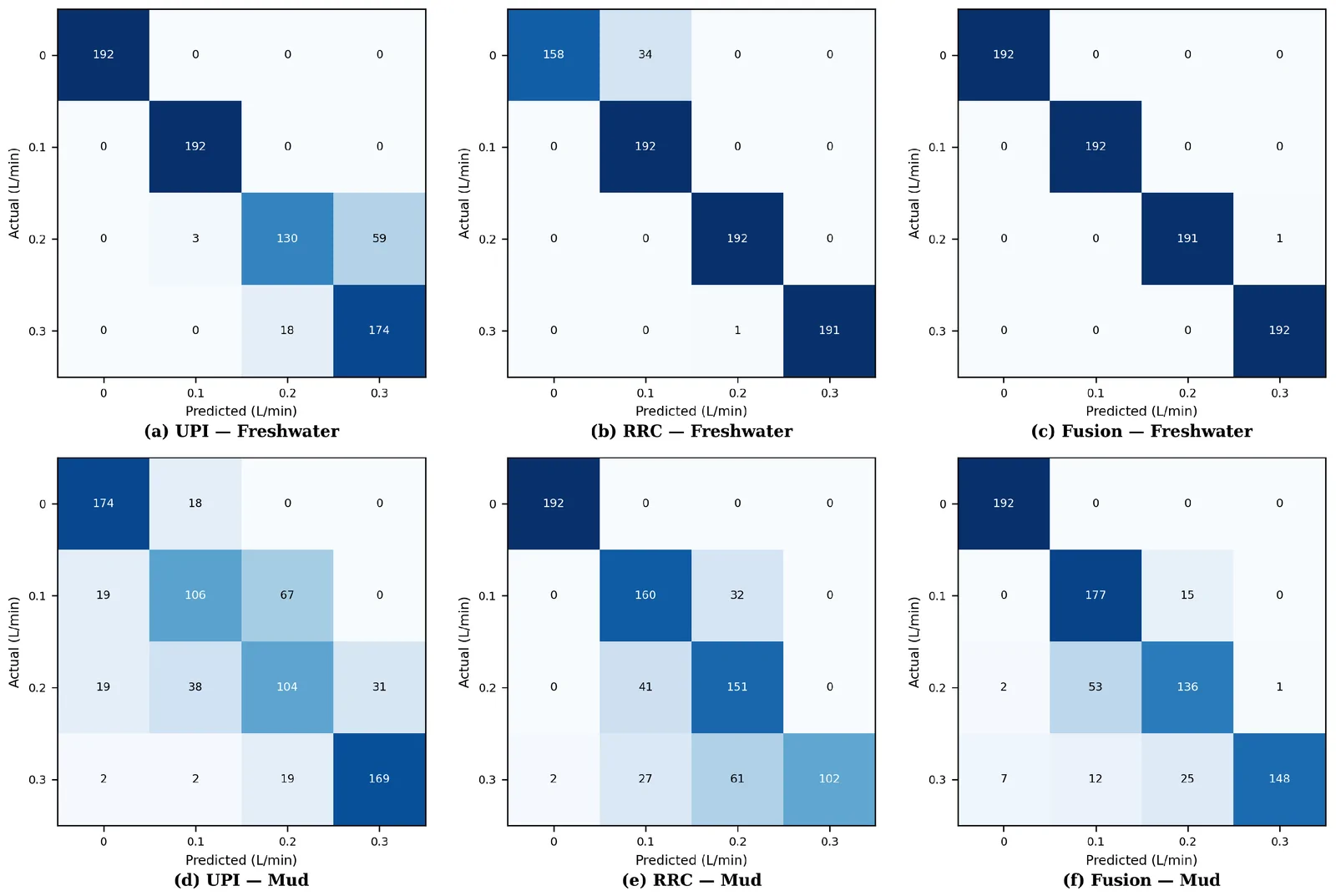
Confusion matrices for window outputs from the ultrasonic (UPI), resistivity (RRC), and fusion methods in freshwater and $2.0\;g\;{\mathrm{cm}}^{-3}$ water-based mud.

Table 4 reports the F1 score for each class and the overall macro F1 score, and Figure 7 shows the corresponding confusion matrices.

In freshwater, misclassifications are confined to adjacent rate classes. Fusion addresses the errors from both single channel failure modes, yielding a macro F1 of 99.9%, an improvement of 4.5 percentage points over the best single channel.

In mud, the confusion is broader. UPI misclassifies samples mostly between neighboring rates. RRC correctly classifies all baseline windows but misclassifies 90 of 192 samples at 0.3 L min^−1^. Fusion increases the number of correct classifications at this rate from 102 to 148. The macro F1 of 84.7% improves on the RRC baseline by 6.3 percentage points. Fusion produced a higher macro F1 than either individual channel in each of the three reserved experimental repetitions for both media.

**Figure 8:**
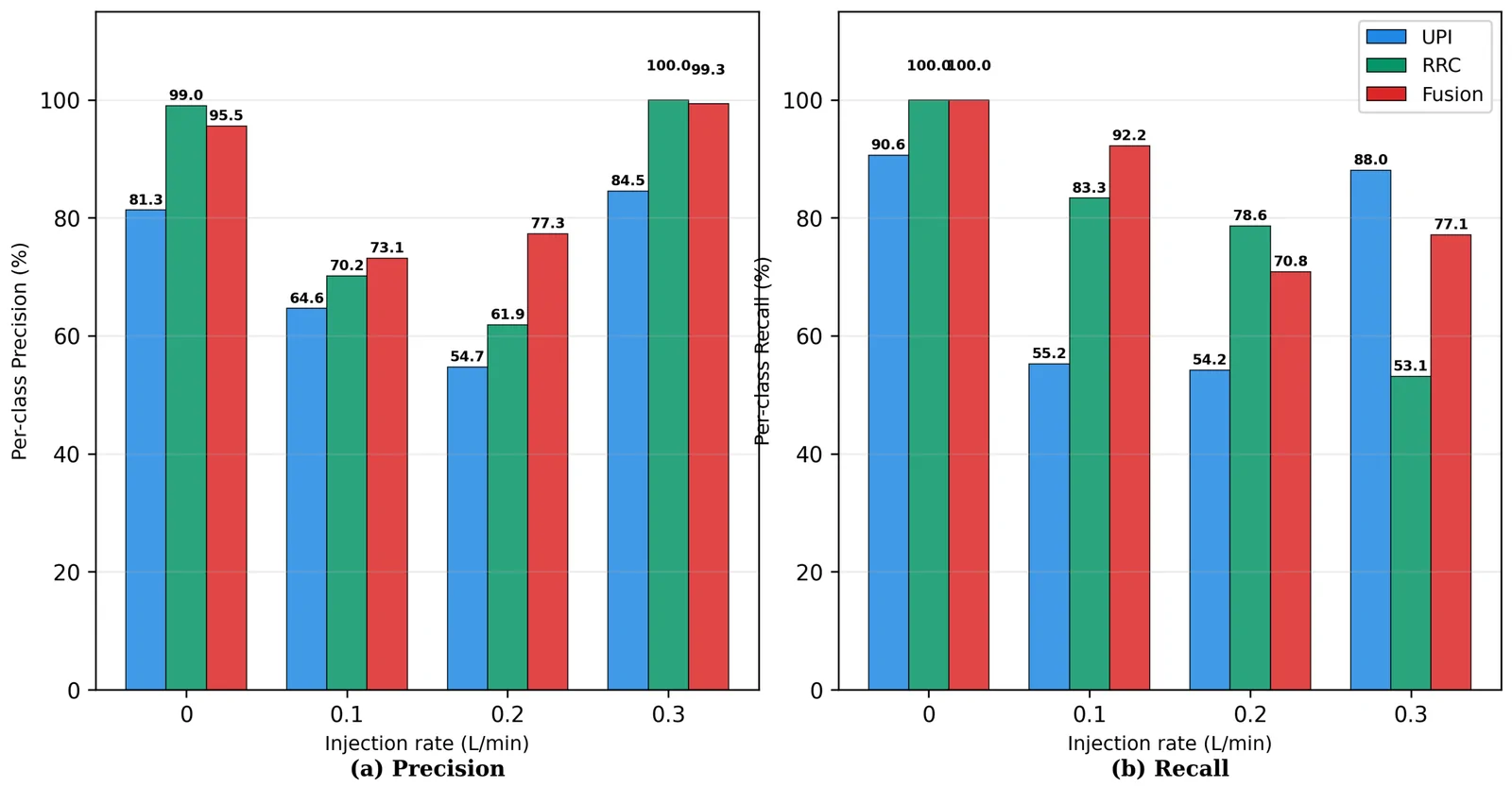
Precision and recall for each injection rate using the ultrasonic (UPI), resistivity (RRC), and fusion methods in $2.0\;g\;{\mathrm{cm}}^{-3}$ water-based mud.

Figure 8 separates the F1 score into precision and recall for each class in mud. At 0.3 L min^−1^, RRC precision reaches 100.0% while recall decreases to 53.1%. The substantial overlap between the RRC distributions at 0.2 and 0.3 L min^−1^ corresponds to limited separation of the two highest rates. UPI maintains higher recall at this rate but at lower precision. Fusion reaches 99.3% precision and increases recall to 77.1%. At 0.1 L min^−1^, fusion recall reaches 92.2% and exceeds both individual channels. From 0.1 to 0.2 and from 0.2 to $0.3\;L\;\min^{-1}$, the median RRC increments decreased from 0.080 to 0.052, while the interquartile range increased from 0.084 to 0.230 at the highest rate. This weaker increment and greater dispersion may reflect changes in the local gas fraction near the electrodes associated with bubble coalescence, slip, or a change in flow pattern. Over the upper rate interval, the UPI median increased by 26.911. The different spatial weighting of the ultrasonic path and the electrode region provides a basis for complementary information. Fusion increased recall from 53.1% for RRC to 77.1%.

**Figure 9:**
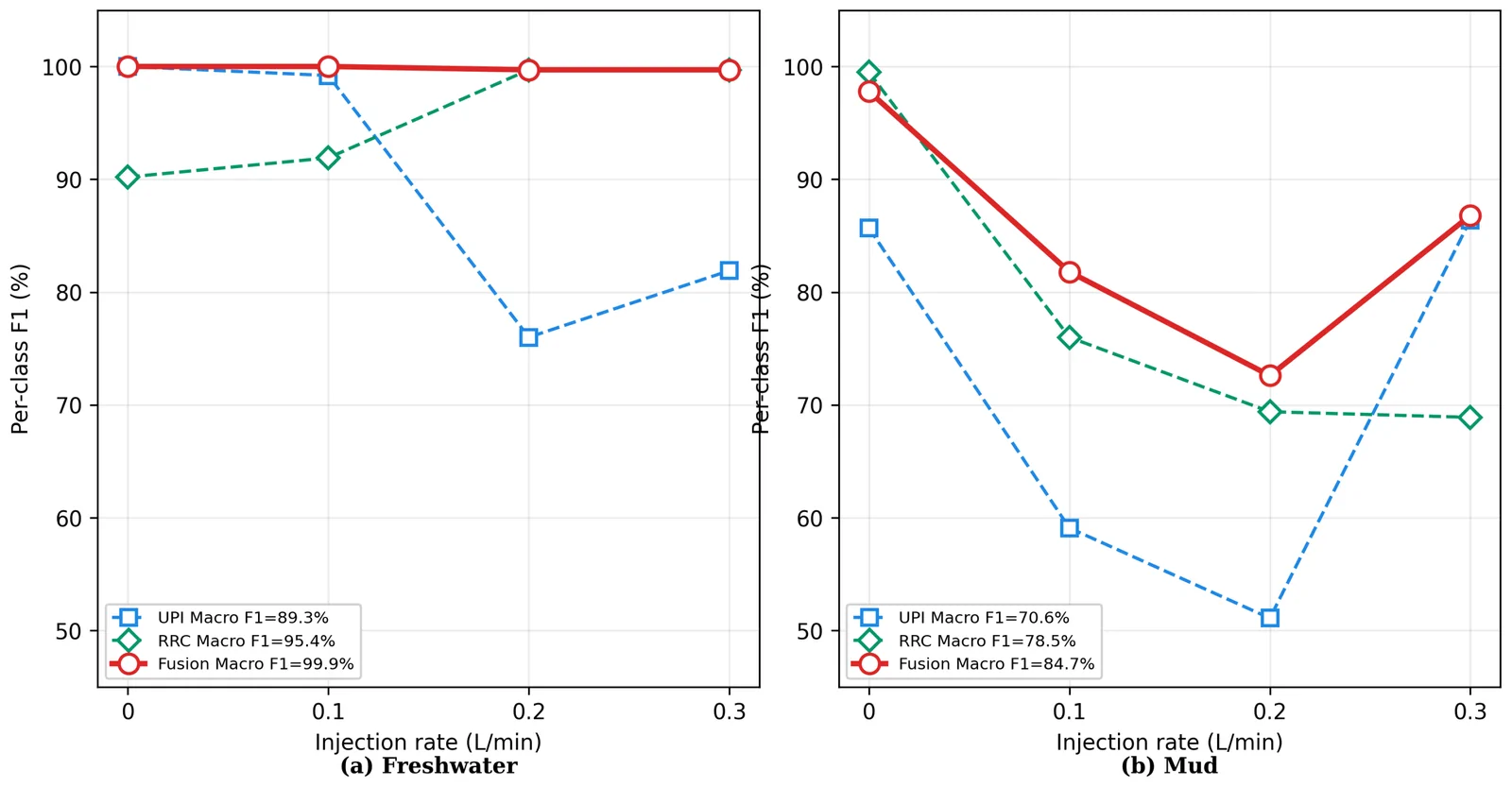
F1 score for each injection rate using the ultrasonic (UPI), resistivity (RRC), and fusion methods in freshwater and $2.0\;g\;{\mathrm{cm}}^{-3}$ water-based mud.

The fusion F1 profile in Figure 9 remains close to the higher of the two individual channel profiles at all four rates in mud. For the fusion output, the remaining errors occur mainly at the 0.1–0.2 and 0.2–0.3 L min^−1^ boundaries, where the response distributions overlap. Related work has likewise used features from the frequency and time-frequency domains for gas–liquid two-phase flow pattern recognition [38].

### 3.4. Effects of Window Aggregation and Sensor Fusion

**Figure 10:**
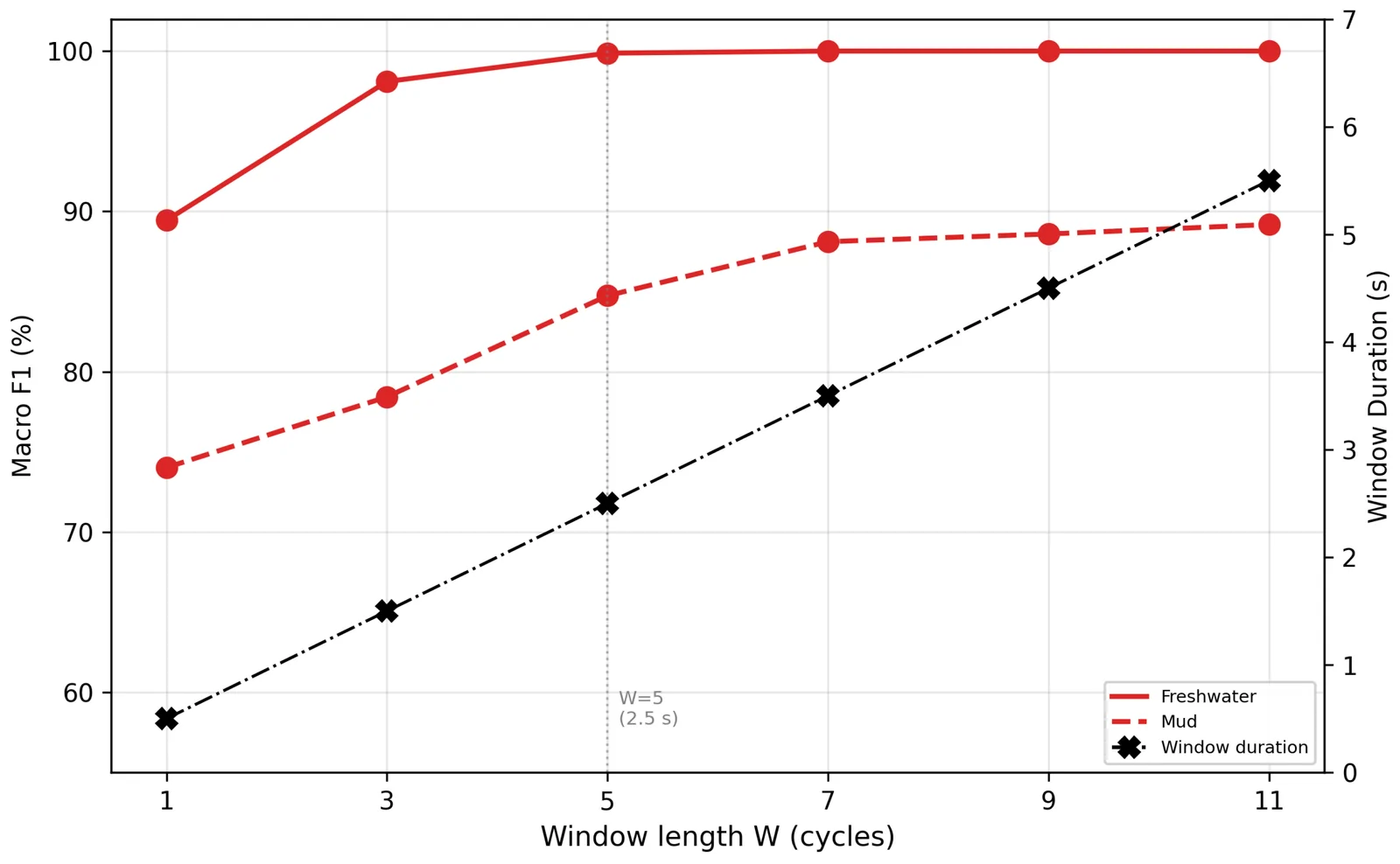
Fusion macro F1 score as a function of window length *W* in freshwater and $2.0\;g\;{\mathrm{cm}}^{-3}$ water-based mud. The secondary axis gives the window duration corresponding to each *W* at two acquisition cycles per second.

Figure 10 examines fusion macro F1 scores across window lengths from W=1 to W=11. In freshwater, the score rises from 89.4% at W=1 to 99.9% at W=5 and then reaches a plateau. In mud, it rises from 74.0% at W=1 to 84.7% at W=5 and continues to 89.2% at W=11. The larger gain in mud is consistent with the broader distributions across acquisition cycles in Figure 6.

The selected 2.5 s window contained five consecutive acquisition cycles, and the one cycle stride produced successive decisions at 0.5 s intervals. Continuous updating is relevant to real time multiphase monitoring [39]. The present experiment evaluated segments recorded after gas injection had stabilized and therefore did not measure the delay between the onset of gas injection and a correct classification. Computation in real time and complete detection latency will be evaluated in future system tests.

Table 5 compares the three nontrainable fusion rules. The classwise product gives the highest observed mean macro F1 in both media. In mud, its advantage over unweighted averaging is 0.70 percentage points, while its advantage over Noisy-OR is 5.07 percentage points. These results support use of the classwise product within the present framework. The comparison is limited to the three evaluated nontrainable fusion rules and does not establish superiority over learnable models.

Table 6 reports six configurations that separate the contributions of window aggregation and fusion of the two channels. M1, M2, and M5 use decisions from individual acquisition cycles, whereas M3, M4, and M6 use W=5 windows.

In freshwater, window aggregation increased the UPI macro F1 by 6.0 percentage points but increased the RRC result by only 0.1 percentage points. RRC already reached 95.3% for individual acquisition cycles, while UPI showed weak separation between 0.2 and $0.3\;L\;\min^{-1}$. In mud, aggregation increased the UPI and RRC results by 12.2 and 12.9 percentage points, respectively. Both channels therefore benefited from aggregation in mud. The difference between media may reflect the distinct spatial weighting of instantaneous bubble configurations along the ultrasonic path and near the probe electrodes.

**Figure 11:**
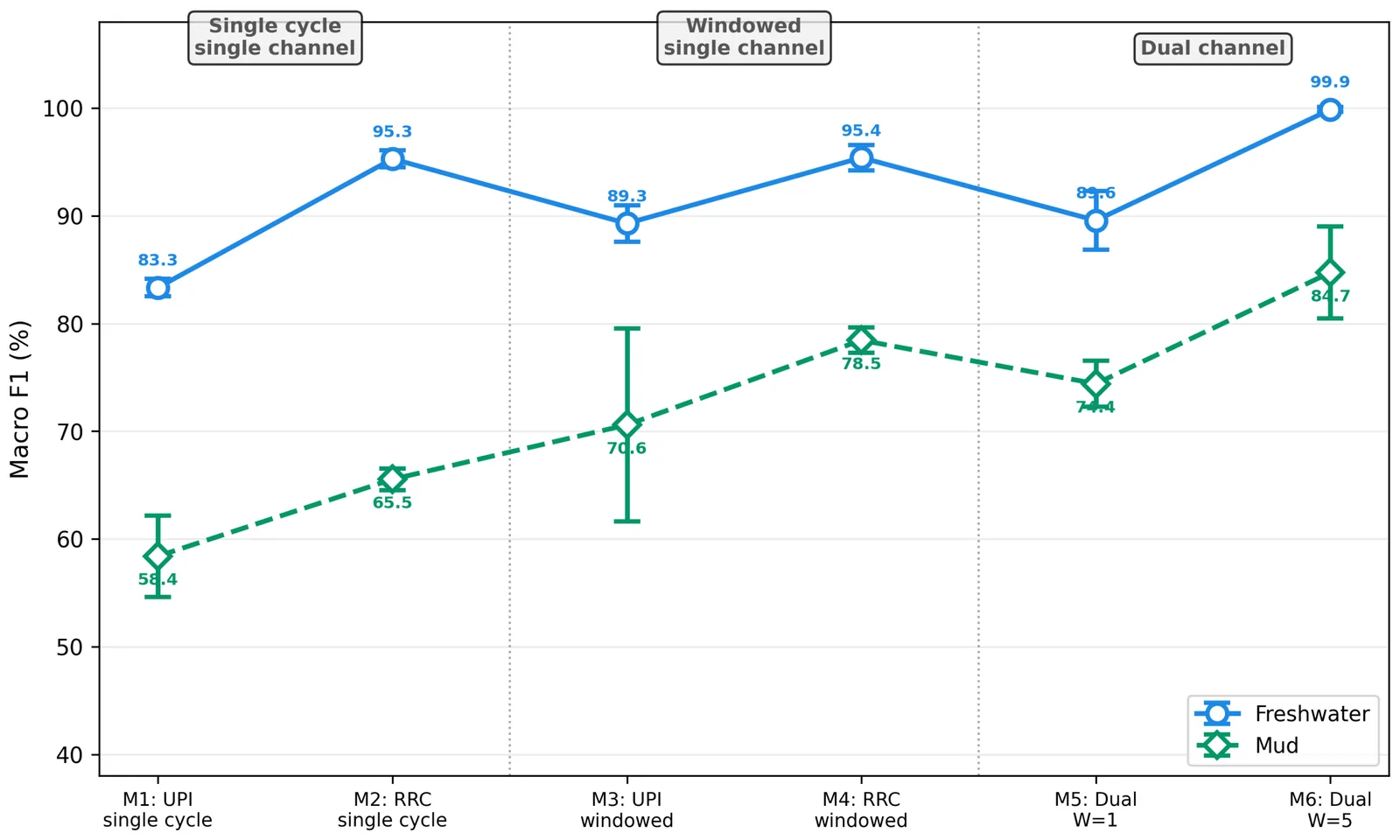
Macro F1 scores for the six ablation configurations based on individual acquisition cycles (M1, M2, and M5) or W=5 windows (M3, M4, and M6).

Before aggregation, combining the channels improves the macro F1 score in mud by 8.9 percentage points over the better individual channel but trails freshwater RRC by 5.7 percentage points. The fused decisions from individual cycles therefore do not preserve the separation achieved by the stronger individual channel in freshwater. With W=5, fusion exceeds the better windowed individual channel by 4.5 percentage points in freshwater and 6.3 percentage points in mud. These gains support a complementary contribution from the two channels after temporal aggregation, as shown in Figure 11.

### 3.5. Mud Circulation Experiment

**Figure 12:**
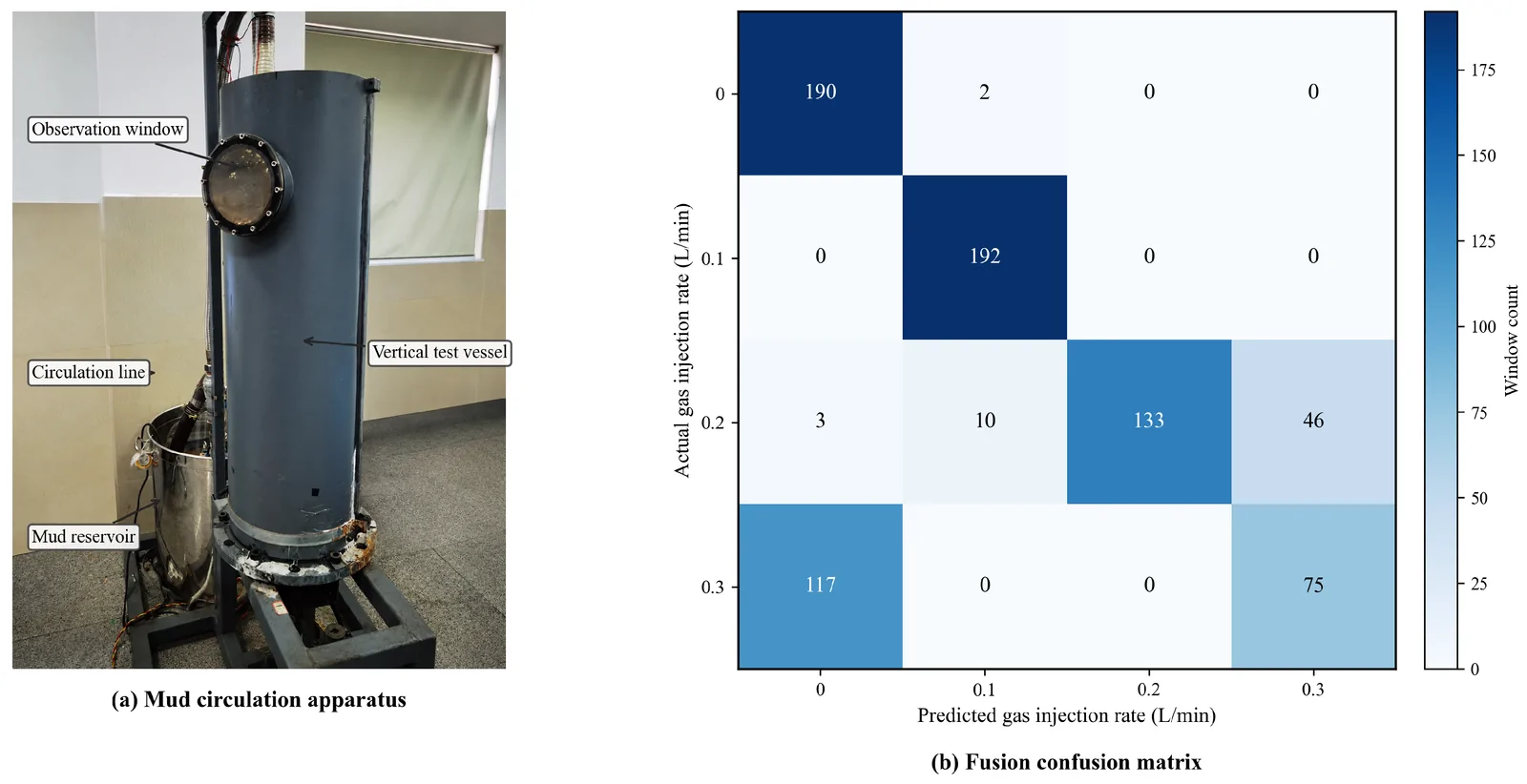
Auxiliary experiment with circulating water-based mud. (**a**) Laboratory circulation apparatus with the principal visible components annotated. (**b**) Aggregate confusion matrix for product fusion at gas injection rates of 0, 0.1, 0.2, and $0.3\;L\;\min^{-1}$.

To examine whether the fusion advantage persists when the mud is circulated, an exploratory experiment was conducted with circulating water-based mud using the apparatus in Figure 12. The same four gas injection rates were used as in the static column experiment. After signal integrity screening, 204 acquisition cycles were retained at each rate and divided chronologically into three sequences of 68 cycles. Applying W=5 separately to each sequence produced 192 windows per rate. One sequence per rate was evaluated in turn using channel thresholds determined from the other two sequences, while the same feature definitions and classwise product fusion rule were retained.

UPI, RRC, and fusion produced macro F1 scores of 36.5%, 63.8%, and 75.6%, respectively, under mud circulation. Fusion exceeded the stronger individual channel by 11.8 percentage points. The two lowest rates were almost completely separated in Figure 12b. Confusion remained at $0.2\;L\;\min^{-1}$ and was strongest at $0.3\;L\;\min^{-1}$, where 117 windows were assigned to the baseline. The fusion advantage observed in the static column was therefore also present under mud circulation, although the highest rate remained difficult to distinguish. The water-based mud was opaque and had high viscosity, and larger bubbles were observed as gas emerged at the liquid surface, although their internal paths and the timing of coalescence could not be observed. Under circulation, advection, shear, pressure fluctuations, and mechanical disturbance may change the bubble distribution relative to the ultrasonic path and the region near the electrodes, increasing variation among acquisition cycles. This interpretation is consistent with the lower macro F1 and the 117 windows assigned to the baseline at $0.3\;L\;\min^{-1}$, but it was not independently verified by bubble imaging or synchronized measurements of flow disturbance.

## 4. Conclusions

To discriminate gas injection rates under intermittent bubble passage conditions, a framework that combines ultrasonic transmission and resistivity measurements with temporal aggregation in a sliding window was developed. UPI and RRC values from individual acquisition cycles were aggregated within the window and fused by normalizing classwise products.

Evaluation by cross-validation with data split by experimental repetition covered freshwater and $2.0\;g\;{\mathrm{cm}}^{-3}$ water-based mud at four injection rates from 0 to $0.3\;L\;\min^{-1}$. The fusion framework attained macro F1 scores of 99.9% and 84.7%, representing gains of 4.5 and 6.3 percentage points over the best result from either channel alone. Temporal aggregation made the larger contribution, while combining the channels provided an additional gain after aggregation.

Among the three evaluated nontrainable fusion rules, the classwise product achieved the highest mean macro F1 in both media. This result supports its use in the present framework. An auxiliary experiment with circulating mud also retained a fusion advantage over both individual channels, although the highest injection rate remained difficult to distinguish. The framework should be further evaluated under representative downhole conditions, particularly high pressure and high temperature, before practical deployment. These results provide a repetition level evaluation of the comparative benefit of fusion under the tested static column conditions. Evaluation across a larger number of independent experiments is required to characterize broader generalization. Future work will examine higher spatial resolution for the electrical measurement through additional electrodes or reduced spacing, together with ultrasonic attenuation spectra as an additional source of information about bubble size.

## Figures and Tables

**Table 1:** Classification thresholds for individual acquisition cycles. Values are the ranges selected across the three cross-validation folds.

Medium	Channel	L1	L2	L3	Unit
Freshwater	UPI	5	60	75	–
Freshwater	RRC	8.5	8.8	9.2	%
Mud	UPI	5	15	30–35	–
Mud	RRC	0.2	0.5	0.6	%

**Table 2:** Fisher discriminant ratio between adjacent gas injection rate classes.

Medium	Channel	0 vs. 0.1	0.1 vs. 0.2	0.2 vs. 0.3
Freshwater	UPI	126.96	10.36	0.68
	RRC	3.76	14.41	7.38
Water-based mud	UPI	1.29	0.19	0.95
	RRC	14.22	0.59	0.08

**Table 3:** Binary detection performance (n=3 folds).

Medium	Method	FAR (Pooled)	Miss (Pooled)	Binary F1 (%)
Freshwater	UPI	0/192 (0%)	0/576 (0%)	100.0 ± 0.0
Freshwater	RRC	34/192 (17.7%)	0/576 (0%)	97.1 ± 0.8
Freshwater	Fusion	0/192 (0%)	0/576 (0%)	100.0 ± 0.0
Mud	UPI	18/192 (9.4%)	40/576 (6.9%)	94.9 ± 3.7
Mud	RRC	0/192 (0%)	2/576 (0.3%)	99.8 ± 0.3
Mud	Fusion	0/192 (0%)	9/576 (1.6%)	99.2 ± 1.0

**Table 4:** Discrimination performance across four injection rates (n=3 folds).

Medium	Method	Rate 0	Rate 0.1	Rate 0.2	Rate 0.3	Macro F1 (%)
Freshwater	UPI	100.0	99.2	76.0	81.9	89.3 ± 1.7
	RRC	90.2	91.9	99.7	99.7	95.4 ± 1.2
	Fusion	100.0	100.0	99.7	99.7	99.9 ± 0.2
Mud	UPI	85.7	59.1	51.1	86.4	70.6 ± 9.0
	RRC	99.5	76.0	69.4	68.9	78.4 ± 1.2
	Fusion	97.8	81.8	72.6	86.8	84.7 ± 4.3

**Table 5:** Comparison of fusion rules using the same cross-validation folds, channel thresholds, and W=5 windows (n=3 folds, macro F1 mean ± standard deviation).

Fusion Rule	Freshwater (%)	Mud (%)
Classwise product	99.87 ± 0.23	84.74 ± 4.28
Unweighted average	98.56 ± 1.13	84.04 ± 3.27
Noisy-OR	98.56 ± 1.13	79.67 ± 3.72

**Table 6:** Ablation matrix (n=3 folds, macro F1 mean ± standard deviation). Configurations based on individual acquisition cycles were evaluated at the 64 decision positions per repetition matched to the W=5 configurations.

Config.	Channel	Aggregation	Fusion	Freshwater	Mud
				Macro F1 (%)	Macro F1 (%)
M1	UPI	Individual cycle	–	83.3 ± 0.8	58.4 ± 3.8
M2	RRC	Individual cycle	–	95.3 ± 0.8	65.5 ± 1.0
M3	UPI	Window, W=5	–	89.3 ± 1.7	70.6 ± 9.0
M4	RRC	Window, W=5	–	95.4 ± 1.2	78.4 ± 1.2
M5	Dual	Individual cycle	Classwise products	89.6 ± 2.7	74.4 ± 2.2
M6	Dual	Window, W=5	Classwise products	99.9 ± 0.2	84.7 ± 4.3

## Data Availability

The raw data supporting the conclusions of this article will be made available by the authors on request.

## Abbreviations

The following abbreviations are used in this manuscript:

UPI	Ultrasonic Propagation Index
RRC	Relative Resistivity Change
TOF	Time of Flight
F1	F1 score (harmonic mean of precision and recall)
